# Nevus of Ota in 8-year-old male

**DOI:** 10.11604/pamj.2023.45.81.40463

**Published:** 2023-06-09

**Authors:** Srilekha Berelli, Sachin Daigavane

**Affiliations:** 1 Department of Ophthalmology, Jawaharlal Nehru Medical College, Datta Meghe Institute of Higher Education and Research, Sawangi (Meghe), Wardha, Maharashtra, India

**Keywords:** Hyperpigmentation, melanin, melanocytosis

## Image in medicine

Occulodermal melanocytosis is also called nevus of Ota. It is a unilateral condition where pigmentation of the conjunctiva and episcleral is seen. It is caused due to increased melanin and melanocytes. These patients have higher chances of having malignant melanoma involving the eye and central nervous system. On slit lamp examination, superficial lesions are grey in colour and deep lesions are blue in colour. On fundus examination we can see hyperpigmented patches of choroid layer with or without optic disc involvement. Ultrasound bio-microscope is a useful imaging technique to see both anterior and posterior segment involvement. Four percent (4%) of patients with unilateral nevus of Ota have chances to develop malignant melanoma in the diseased eye. It is important to do dilated fundus examination in both eyes every six months along with systemic evaluation. Here is a case of 8-year-old male patient with complaints of discolouration in right eye since childhood. There is no dermal involvement at present. The patient gives 6/6 vision on Snellen's chart in both eyes. Hyperpigmented greyish colour areas are present in bulbar conjunctiva and episcleral patches present indicate high melanin and melanocytes. As there is 4% chance of developing choroidal melanoma, the patient was advised to follow-up for every 6 months for detailed ocular examination and systemic evaluation.

**Figure 1:**
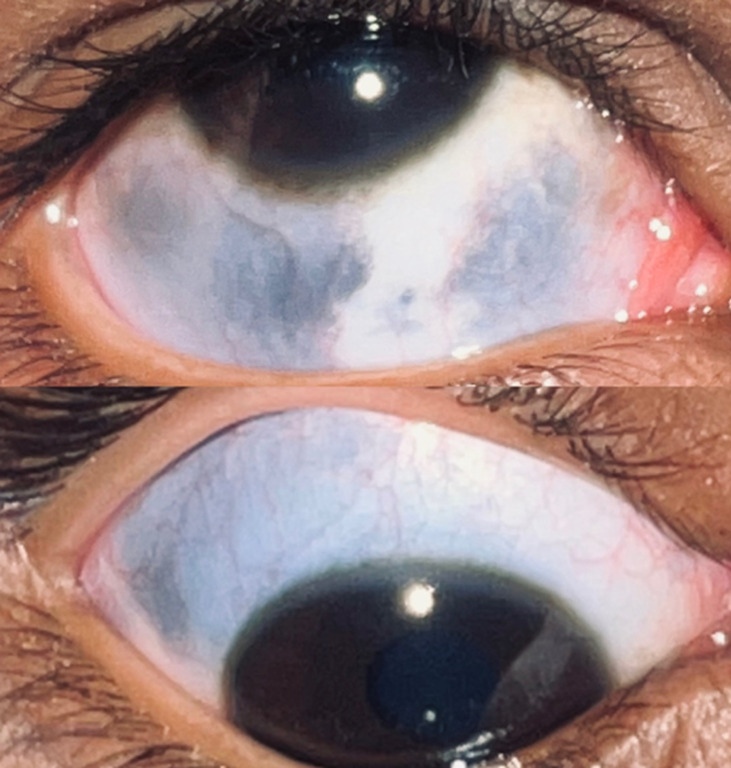
nevus of Ota in the right eye

